# Graphsene as a novel porous two-dimensional carbon material for enhanced oxygen reduction electrocatalysis

**DOI:** 10.1038/s41598-024-59756-3

**Published:** 2024-04-21

**Authors:** Mohammadreza Hosseini, Maryam Soleimani, Fazel Shojaei, Mahdi Pourfath

**Affiliations:** 1 Department of Physical Chemistry, Tarbiat Modares University, Tehran, Iran; 2 School of Electrical and Computer Engineering, University of Tehran, Tehran, 14395-515 Iran; 3 Department of Chemistry, Faculty of Nano and Bioscience and Technology, Persian Gulf University, Bushehr, 75169 Iran; 4 Institute for Microelectronics, TU Wien, Gußhausstraße 27-29, A-1040 Vienna, Austria

**Keywords:** Graphene allotropes, Porous carbon-based materials, Two-dimensional materials, Oxygen reduction reaction electrocatalysis, Materials science, Condensed-matter physics, Materials for energy and catalysis, Nanoscale materials

## Abstract

Graphene allotropes with varied carbon configurations have attracted significant attention for their unique properties and chemical activities. This study introduces a novel two-dimensional carbon-based material, termed Graphsene (GrS), through theoretical study. Comprising tetra-, penta-, and dodeca-carbon rings, GrS’s cohesive energy calculations demonstrate its superior structural stability over existing graphene allotropes, including graphyne and graphdiyne families. Phonon dispersion analysis confirms GrS’s dynamic stability and its relatively low thermal conductivity. All calculated GrS elastic constants meet the Born criteria, ensuring mechanical stability. Ab-initio molecular dynamic simulations show GrS maintains its structure at 300 K. HSE06 calculations reveal a narrow electronic bandgap of 20 meV, with the electronic band structure featuring a highly anisotropic Dirac-like cone due to its intrinsic structural anisotropy along armchair and zigzag directions. Notably, GrS is predicted to offer exceptional catalytic performance for the oxygen reduction reaction, favoring the four-electron reduction pathway with high selectivity under both acidic and alkaline conditions. This discovery opens promising avenues for developing metal-free catalyst materials in clean energy production.

## Introduction

In the quest for versatile two-dimensional (2D) carbon-based materials, researchers have explored beyond graphene, delving into non-benzenoid nanosheets^1,2^ with unique electronic, mechanical, and optical properties. Despite graphene's remarkable attributes, its zero bandgap and chemical inertness pose limitations for certain applications^3–5^. Addressing these challenges, structural modifications and the exploration of different crystal systems have yielded a variety of planar surfaces characterized by carbon rings of various sizes, each promising distinct electronic, optical, and mechanical advantages^6,7^. These materials are characterized by the presence of carbon rings of varying sizes. The search for new carbon-based materials with desired characteristics is an active field in physics, chemistry, and materials science^8,9^.

Experimental advancements in the synthesis of carbon nanosheets, characterized by adjustable porosity and diverse surface morphologies, have been achieved^10,11^. Complementing these successes, theoretical research opens avenues for the discovery of functional materials. An example includes the conceptualization of biphenylene (BP), envisioned as a carbon monolayer amalgamating 4-, 6-, and 8-carbon rings to exhibit metallic properties^12^. Subsequently, Fan et al. synthesized BP through a bottom-up methodology, validating its metallic nature via scanning probe characterization^13^. This was further bolstered by theoretical studies, suggesting BP’s potential in applications such as gas sensing^14^ and as ion-battery anode material^15^. The crystallization of BP into a hexagonal system introduced graphenylene, a monolayer with a notably narrow electronic band gap^16^. Additionally, Wang et al. identified phagraphene, an allotrope of graphene distinguished by a distorted Dirac cone, identified through a systematic evolutionary algorithm^17^. This exploration extended to the unveiling of T-graphene, arising from the periodic configuration of tetragonal carbon rings, deviating from the conventional hexagonal lattice and featuring Dirac-like fermions with linear dispersion^18^. Intriguingly, the insertion of varied elements into T-graphene’s lattice has been shown to modify its electronic attributes significantly^19^.

Within the realm of 2D carbon-based materials, Graphyne and Graphdiyne stand out for their unique combination of perfect hexagonal symmetry and semiconducting behavior, characterized by hybridizations of *sp* and *sp*^2^ carbon atoms^20–22^. These materials have captured the attention of researchers due to their potential in enhancing the efficiency of Na-ion batteries as anode materials and in finely tuning thermoelectric properties for energy conversion applications^23,24^. In a significant advancement, the recent synthesis of holey-graphyne has marked a pivotal development. This functional porous material, with an electronic band gap energy of 1.1 eV, not only exhibits a high thermoelectric figure of merit (ZT = 1.5) at room temperature but also demonstrates enhanced hydrogen evolution reaction (HER) performance^25,26^.

Penta-graphene, a pioneering carbon-based nanosheet composed entirely of pentagons^27^, has been theoretically predicted to exhibit stability with a notable 3.25 eV electronic band gap. This innovative nanostructure was designed through the exfoliation of a single-layer carbon from the T-12 carbonaceous compound. Following this, Haeckelite emerges as another planar carbon-based material, distinguished by its 5-, 6-, and 7-membered rings, which confer it with electrical conductivity and mechanical properties on par with graphene^28^. The unveiling of tetra-penta-octagonal graphene (TPO-graphene) further expands the structural diversity, revealing a unique integration of tetragons and pentagons linked by octagon formations^29^. Subsequently, Yu et al. introduced QPHT-graphene, a novel 2D carbon-based nanosheet incorporating an intricate arrangement of 4-, 5-, 6-, and 14-carbon ring fragments^30^. The high density of states (DOS) at the Fermi level signifies its metallic characteristics, underscoring the potential for diverse energy storage applications. The concept of structural rearrangement brings functionality to graphene allotrope family members^31^. For example, Popgraphene, which is recognized by its 5–8–5 carbon ring configuration, demonstrates exceptional performance as an anode material in Lithium-ion batteries^32^. Moreover, density functional theory (DFT) calculations highlight biphenylene's superior electrocatalytic activity for the HER compared to graphene, marking a significant advancement in electrocatalysis^33^.

In this study, Graphsene (GrS) is introduced as a new member to the 2D carbon-based materials family, uniquely characterized by its tetra-, penta-, and dodeca-carbon rings structure. A comprehensive assessment of GrS's mechanical, dynamical, and thermal properties confirms its robust stability as a hypothetical material with feasible experimental synthesis. Utilizing density functional theory (DFT) calculations, it is determined that GrS exhibits the characteristics of a narrow bandgap semiconductor. This, coupled with its notable porosity and electrical conductivity, positions Graphsene as a highly promising material for catalytic applications, particularly in enhancing the efficiency of the oxygen reduction reaction (ORR).

## Method

To accurately characterize the structural, electronic, and catalytic properties of GrS, DFT calculations were employed, as facilitated by the Vienna Ab Initio Simulation Package (VASP)^34^. We initiated our computational exploration with the Perdew–Burke–Ernzerhof (PBE) functional, under the generalized gradient approximation (GGA) framework, complemented by the projector augmented-wave (PAW) method to effectively model exchange–correlation effects. The electronic band structure was carefully analyzed using the HSE06 hybrid functional which is crucial for accurately determining electronic characteristics of GrS.

Optimizations of the wavefunctions were achieved through a plane wave basis set, specified with a cutoff energy of 700 eV. For structural relaxation, encompassing atomic positions and lattice vectors, we utilized a 15 × 15 × 1 grid to sample the Brillouin zone, while a denser 25 × 25 × 1 grid, following the Monkhorst–Pack algorithm, was applied for electronic charge density calculations. The rectangular cell's Brillouin zone was sampled with a 17 × 25 × 1 grid during electronic density calculation. For both structures, a 20 Å vacuum along the z-direction was incorporated to avoid interlayer interactions.

Ensuring convergence and stability, the self-consistent field (SCF) calculations and structural optimizations adhered to an energy convergence criterion of 10^–8^ eV. The dynamical stability of GrS was verified via phonon dispersion spectrum analysis, employing the density functional perturbation theory (DFPT) approach within the Phonopy code^35^. Ab initio molecular dynamics (AIMD) simulations at 300 K over 10 ps, conducted on a 4 × 4 supercell, affirmed the thermal stability of GrS.

Mechanical stability was investigated through the calculation of elastic constants for a rectangular system with an orthorhombic crystal lattice, utilizing vaspkit for post-processing tool^36^. To elucidate the catalytic efficacy of GrS for ORR catalysis, we adopted the computational hydrogen electrode (CHE) model, proposed by Nørskov et al. The Gibbs free energy change (ΔG) for each ORR step was obtained by:

$$\Delta {\text{G }} = \, \Delta {\text{E }} - {\text{ T}}\Delta {\text{S }} + \, \Delta {\text{ZPE}}$$

where ΔE is the electronic energy difference derived directly from DFT calculations, ΔZPE represents the change in zero-point energy (ZPE), and TΔS accounts for the change in entropy at 298.15 K. The ZPE and entropies of ORR intermediates were assessed through vibrational frequency calculations. For standard molecules such as H_2_ and H_2_O, their entropic values are sourced from the reputable NIST database to ensure accuracy. To address the limitations of DFT in describing the high-spin ground state of O_2_, the Gibbs free energy of O_2_ (GO_2_) is calculated as follows:

$${\text{G}}_{{{\text{O}_{2}}}} = {\text{ 2G}}_{{{\text{H}_{2}\text{O}}}} - {\text{ 2G}}_{{{\text{H}_{2}}}} + { 4.92}\,{\text{eV}}.$$

The effects of pH and the electrode potential (U) on the O_2_ reduction reaction are considered as energy shifts in the free energy changes during electrochemical steps: ΔG_pH_ =  − k_B_Tln10 × pH and ΔG_U_ =  − neU, where k_B_ is the Boltzmann constant, e is the elementary charge, and n is the number of transferred proton-electron pairs. To assess the thermodynamic activity of the ORR, with a four-electron reaction mechanism, the limiting potential of the reaction (U_L_) can be determined by:

$${\text{U}}_{{\text{L}}} = \, - {\text{max}}\left\{ {\Delta {\text{G}}_{{1}} , \, \Delta {\text{G}}_{{2}} , \, \Delta {\text{G}}_{{3}} , \, \Delta {\text{G}}_{{4}} } \right\}/{\text{e}}$$

where ΔG_n_ (n = 1–4) is the reaction free energy of the nth electrochemical step. Within this framework, a higher limiting potential (U_L_) value is indicative of superior catalytic performance.

## Results and discussion

### Structural analysis

**Figure 1:**
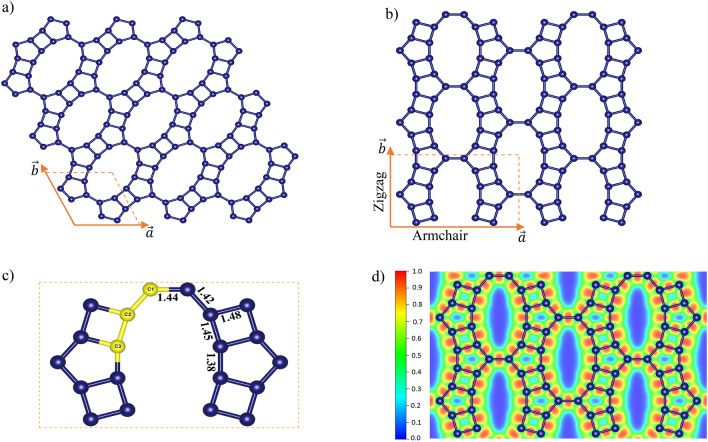
Schematic presentation of (**a**) rectangular and (**b**) primitive unit cell of GrS. (**c**) distinct carbon atoms and C–C bonds in GrS structure. (**d**) In-plane ELF map of GrS.

Figure 1 provides a detailed representation of both the primitive cell and the smallest rectangular supercells of GrS, illustrating the meticulous optimized atomic arrangement of GrS. Accordingly, for the primitive cell, the lattice parameters are a = b = 5.96 Å, and γ = 115.15°, featuring an arrangement of 10 carbon atoms in two coupled pentagon rings. The rectangular supercell contains 20 carbon atoms, with orthogonal lattice constants of a = 10.07 Å and b = 6.40 Å. The calculated in-plane carbon density of GrS, 0.31 carbon/Å^2^, exceeds that of the graphyne family (0.19–0.27 carbon/Å^2^), as experimentally synthesized, yet does not reach the densities of graphene (0.38 carbon/Å^2^) or phagraphene (0.37 carbon/Å^2^)^17^. Notably, the GrS structure, as depicted in Fig. 1c, identifies three distinct carbon atoms (C1, C2, and C3) and highlights five types of C–C covalent bonds, with lengths ranging from 1.38 to 1.48 Å. These structural intricacies are consistent with those observed in previous studies of carbonaceous monolayers, suggesting unique mechanical and electronic properties of the studied material. GrS exhibits distinctive porosity, a direct result of its unique structural composition involving the sharing of 4-, 5-, and 12-carbon rings. This configuration not only contributes to its porosity but also imparts a pronounced anisotropic character to the monolayer.

To elucidate the nature of the chemical bonds in GrS, the electron localization function (ELF) was employed to map electron distribution within the plane of this two-dimensional material, as depicted in Fig. 1d. This analysis offers a visual representation of electron density around the various carbon–carbon (C–C) bonds in GrS. The ELF analysis reveals a high degree of electron localization in these bonds, indicating robust covalent interactions between adjacent carbon atoms. The presence of such pronounced covalent bonding implies significant implications for GrS's mechanical strength and chemical stability, underscoring its potential as a resilient material in various applications.

To evaluate the thermodynamic stability of GrS, we evaluated its cohesive energy, which measures the energy required to disassemble the compound into its individual atoms. The calculated cohesive energy for GrS is − 8.48 eV/atom, a value that is comparable to those of other notable carbonaceous materials such as α-graphyne (− 8.30 eV/atom), β-graphyne (− 8.38 eV/atom), phagraphene (− 9.03 eV/atom), and biphenylene (− 7.55 eV/atom)^17,33^. This comparison highlights GrS’s competitive stability, positioning it favorably against some of its predecessors. Furthermore, the relationship between atomic density and thermodynamic stability, previously established in the literature, is reaffirmed by our findings^17^. As a result, based on its cohesive energy, Graphsene (GrS) can be regarded as a stable material from an energy standpoint.

### Stability

Evaluating the dynamical, mechanical, and thermal stability of an unknown material is crucial for understanding its synthesis feasibility and potential applications. Mechanical stability describes a material's resilience to external forces and its ability to retain its structure without deformation. To assess the mechanical stability and elastic properties of GrS, the second-order elastic stiffness tensor (*C*_*ij*_) was calculated for its smallest rectangular unit cell and listed in Table S1. This tensor serves as a quantitative measure of the material's resistance to deformation, with specific criteria required for confirming mechanical stability. The determining elements are presented in Table 1. Accordingly, GrS meets the elastic stability conditions outlined by Coudert et al.^37^: ${C}_{11}{C}_{22}-{C}_{12}^{2}>0$, ${C}_{44}>0$, and ${C}_{66}>0$. These conditions, derived from the Born elastic criteria, confirm that GrS is mechanically stable.

**Table 1:** The stiffness tensor elements corresponding to the rectangular cell in the unit of GPa.

C_11_	C_12_	C_21_	C_22_	C_44_	C_66_
79.51	21.08	21.08	187.36	0.14	27.58

**Figure 2:**
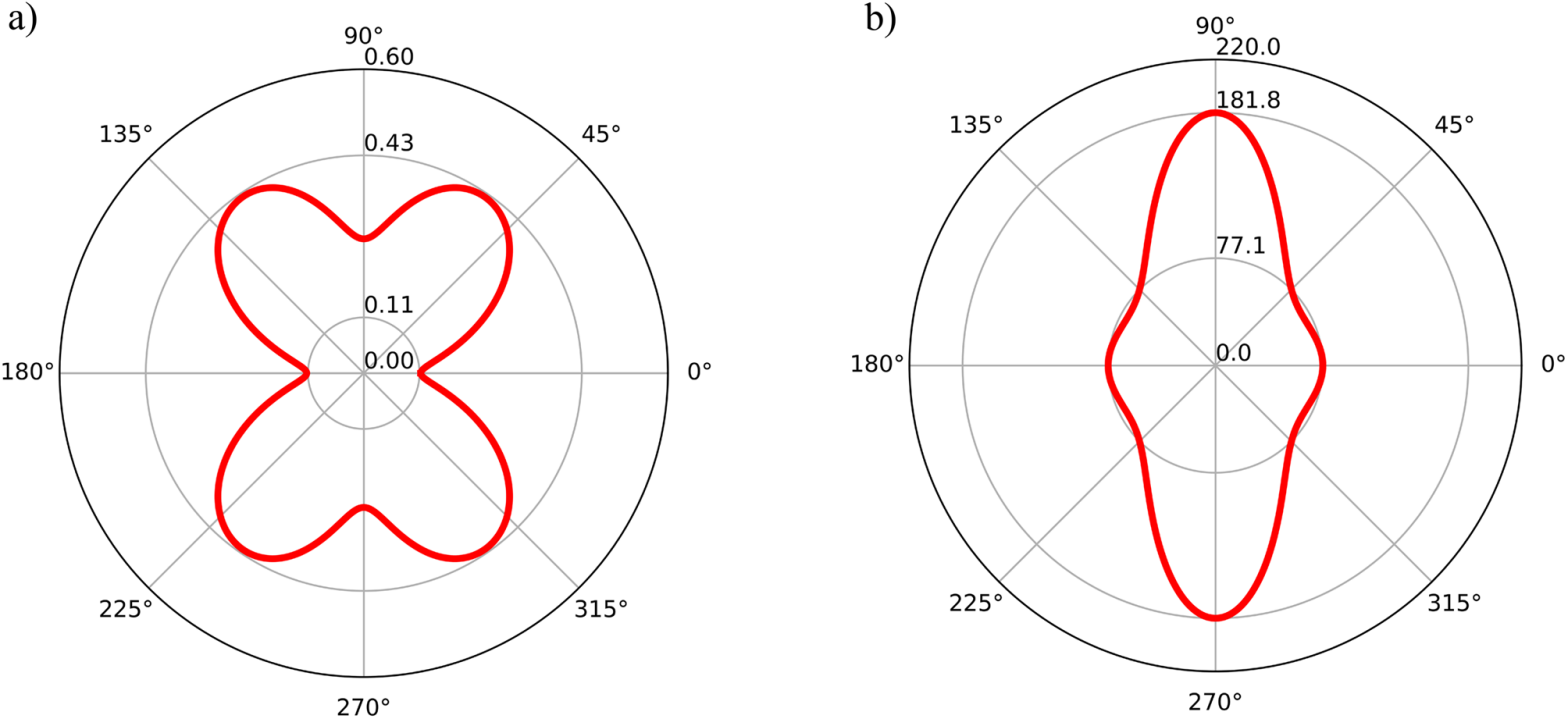
Orientation dependency of (**a**) Poisson’s ratio and (**b**) Young’s modulus of GrS.

The stiffness elements of GrS reveal anisotropic mechanical behaviors, consistent with its distinct atomic arrangements along the zigzag (*y*) and armchair (*x*) directions. For a more detailed examination of GrS’s mechanical properties, the orientation dependence of Young’s modulus, *E(θ)*, and Poisson’s ratio, *ν(θ)*, were assessed using equations (S1) and (S2), with results illustrated in Fig. 2. The polar diagrams clearly demonstrate the anisotropic nature of GrS's mechanical properties within the *x–y* plane. Remarkably, Young’s modulus values in the *x*-direction (*E*_*x*_ = 181.8 GPa) and *y*-direction (*E*_*y*_ = 77.1 GPa) are significantly lower than those reported for graphene (2.4 TPa)^38^, with GrS exhibiting particularly lower stiffness in the armchair (*y*) direction, indicating increased flexibility. Additionally, Poisson’s ratios in the *x*- and *y*-directions were found to be 0.11 and 0.26, respectively, both lower than those observed for biphenylene; 0.38 and 0.31 for x and y directions respectively^33^. Following the methodology of Sun et al. in their DFT-based study on graphene allotropes, GrS's mechanical characteristics categorize it as a notably flexible material within the graphene family^39^.

**Figure 3:**
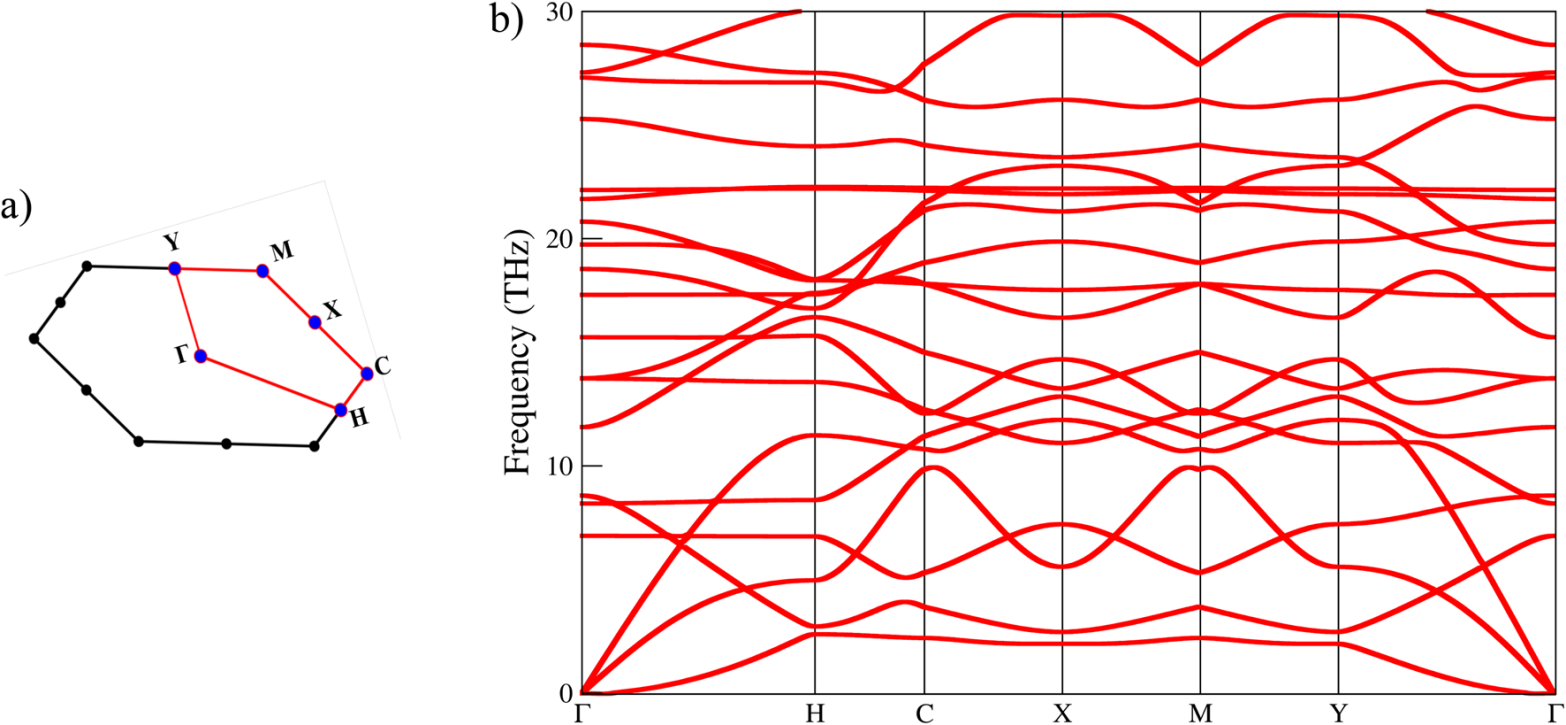
(**a**) First Brillouin zone of primitive unit cell of GrS (**b**) Phonon dispersion spectrum of GrS.

The dynamical stability of GrS was rigorously examined through phonon dispersion calculations. Because of deviation from hexagonal Bravais lattice, the primitive unit cell of GrS introduces additional high-symmetry points in its first Brillouin zone, as illustrated in Fig. 3a. Figure 3b presents the phonon dispersion curves for GrS, mapped along these identified high-symmetry points. The absence of imaginary frequencies within the phonon band structure is a key indicator of GrS’s dynamical stability, highlighting that the material does not exhibit unstable vibrational modes. Notably, the emergence of the first optical phonon mode at a low frequency of 6.92 THz, within the acoustic region, suggests relatively low thermal conductivity. This characteristic is attributed to GrS’s substantial porosity, which disrupts phonon propagation and contributes to its unique thermal properties.

**Figure 4:**
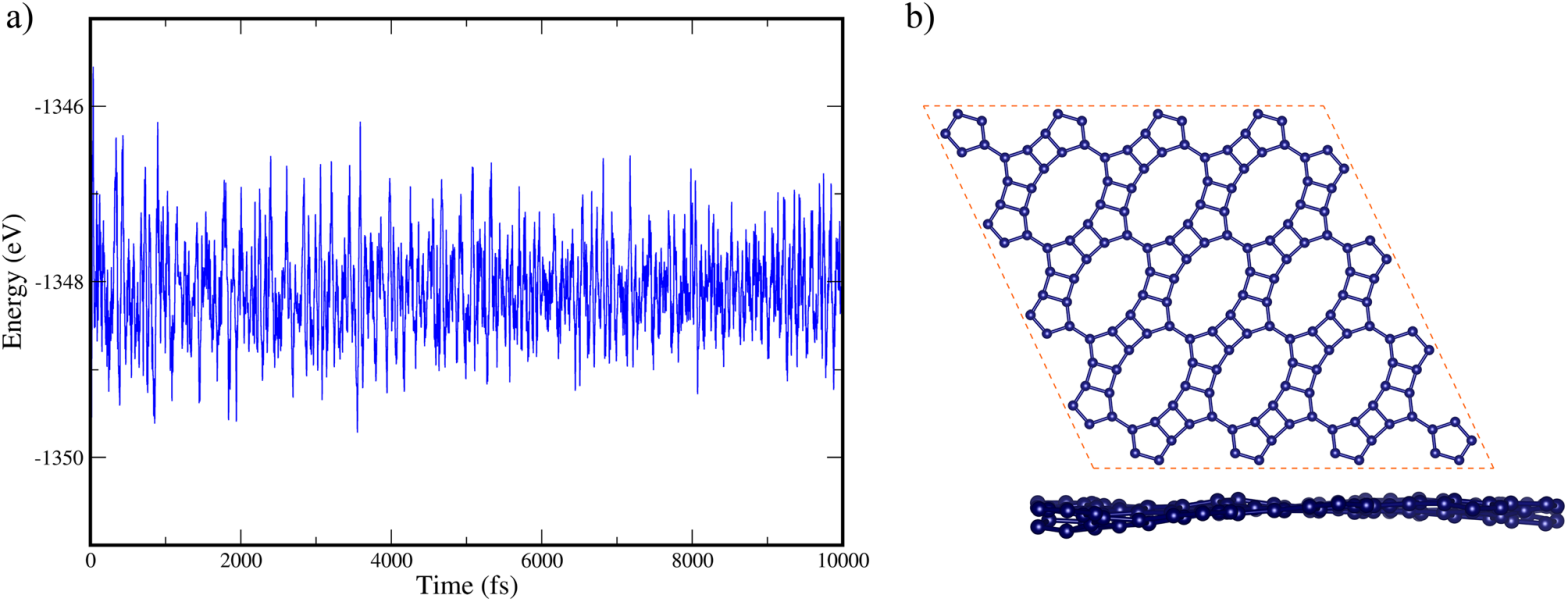
(**a**) AIMD trajectories of free energy and (**b**) Both side and top views of GrS's atomic structure at 300 K.

To evaluate the thermal stability of GrS at room temperature, AIMD simulations were employed. To assess GrS's thermal stability specifically, we performed AIMD calculations at 300 K. The outcomes of these simulations are twofold: the free energy profile (Fig. 4a), serving as a critical quantitative measure of stability by indicating the energy landscape the material experiences under thermal conditions, and the geometry of GrS (Fig. 4b), which showcases its structural integrity when subjected to room temperature. Together, these figures underscore GrS’s robust thermal stability at room temperature.

The presented results underline GrS’s dynamical, mechanical, and thermal stability, crucial properties that facilitate its synthesis. The unchanged geometric parameters at the studied temperature not only affirm the material's robustness but also indicate that GrS can withstand the conditions commonly encountered in synthetic processes. Consequently, these stability attributes open the door for exploring synthetic routes aimed at the successful fabrication of GrS.

### Electronic properties

Given the correlation between electronic and structural properties of materials, it is reasonable to anticipate a diverse range of electronic characteristics in graphene allotropes, including metallic, semi-metallic, and semiconducting behaviors^29,32^. Similarly, for the mechanical properties, employing a rectangular system allows effective incorporation and study of direction-dependent electronic characteristics of GrS. Both the band-structure of BPE and HSE06 perspectives were utilized to conduct a comprehensive study of the electronic properties of the proposed nanostructure.

**Figure 5:**
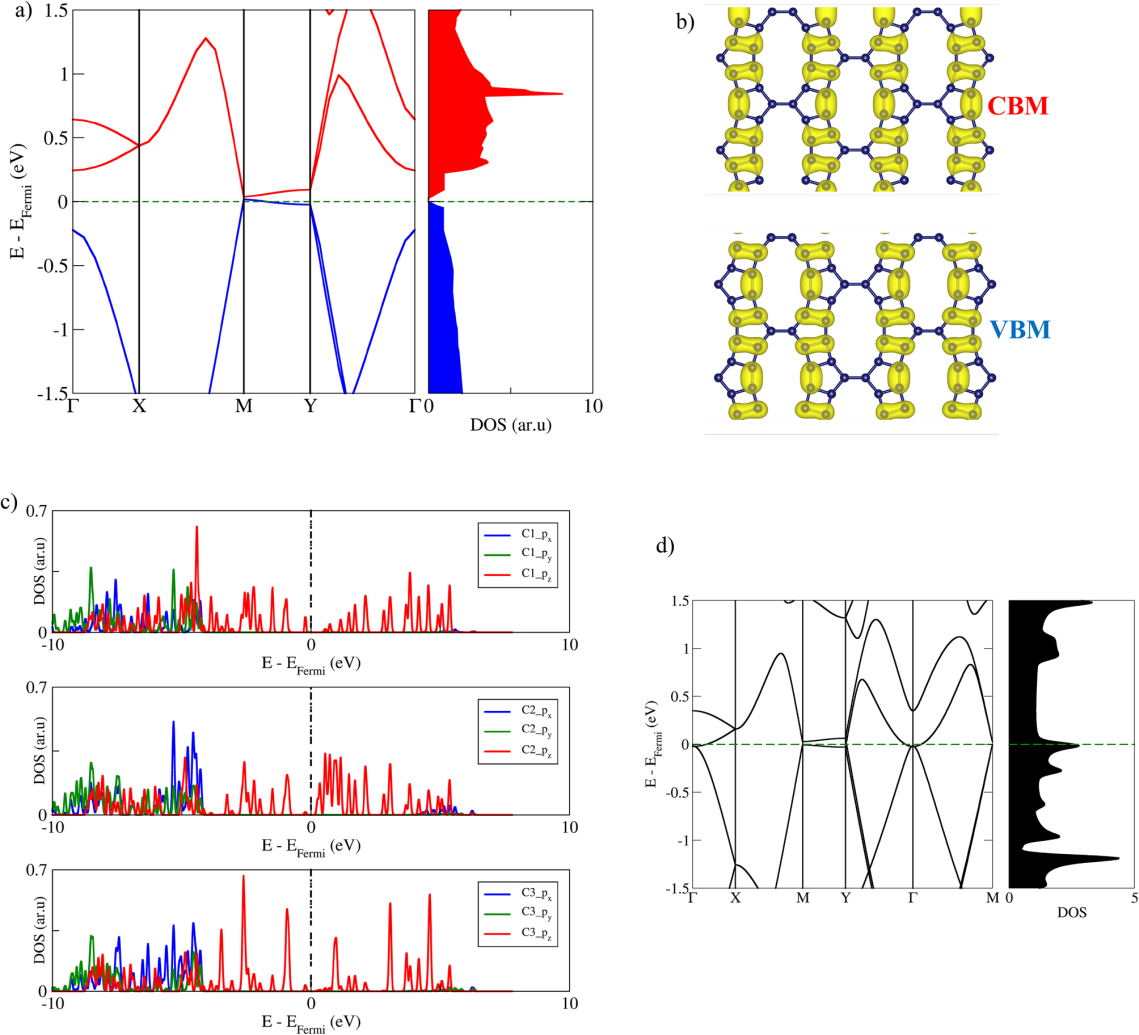
Comprehensive electronic properties of GrS (**a**) HSE06 electronic band structure and DOS of GrS. (**b**) Electronic distribution at the VBM and CBM in GrS (**c**) HSE06 PDOS plots of GrS (**d**) PBE electronic band structure and DOS of GrS.

In the context of the PBE approach, the obtained electronic band structure reveals that certain electronic bands cross the Fermi level, implying metallicity in GrS, as depicted in Fig. 5d. Notably, these bands involve paths in which the Γ point contributes. The results based on HSE06, presented in Fig. 5a, predict a small electronic band gap of 20 meV at the M high-symmetry point. Since this gap is smaller than the thermal energy at room temperature, high electrical conductivity is anticipated for this condition.

Figure 5a demonstrates that both the valence band maximum (VBM) and conduction band minimum (CBM) exhibit a linear dispersion of electronic bands, resulting in the formation of a Dirac-like cone at the M point. Along the M-Y path, associated with the armchair direction, nearly localized states can be observed for both the valence and conduction bands. Conversely, along the X-M path, which follows the zigzag direction, electronic states are more dispersive. To further explore this anisotropy, the Fermi velocities of the frontier bands are calculated and listed in Table 2. It is noteworthy that the velocity along the zigzag direction is significantly higher than that in the armchair direction, evidencing the anisotropic behavior of the electronic properties of GrS. Similar behavior was reported in other carbon-based nanosheets with varied carbon-ring sizes^40^.

**Table 2:** Fermi velocity of conduction and valence bands around the Fermi level in the unit of m/s.

Direction	VBM	CBM
X-M (Zigzag)	9.1 × 10^6^	8.8 × 10^6^
M-Y (Armchair)	1.6 × 10^5^	1.9 × 10^5^

The charge density distribution for the VBM and CBM is depicted in Fig. 5b, showing a significant contribution from carbon atoms C2 and C3 to the material's frontier crystal orbitals. Unlike C1, these carbons exhibit deviations from the ideal *sp*^2^ hybridization, a configuration known for its planar and electron-delocalizing characteristics, which in turn influences the material's electronic activity due to their strained configurations. The analysis indicates that the frontier orbitals are primarily aligned along the zigzag direction, a characteristic that correlates with the previously noted presence of more significant dispersive electronic bands in this orientation. Further insights from Partial Density of States (PDOS) plots, as shown in Fig. 5c, confirms the significant contribution of C2 and C3 near the Fermi level, reinforcing their roles in defining the material's electronic behavior. Consistent with previously reported findings, the *p*_*z*_ orbitals, which are key in forming π-type frontier states, play a central role in determining the electronic properties of in-plane extended carbonaceous materials^41,42^. Additionally, the *p*_*x*_ and *p*_*y*_ orbitals contribute to the material's deeper electronic bands through the hybridization with σ bonds, highlighting the complex interplay of orbital contributions to the overall electronic structure.

### Electrocatalytic activity

**Figure 6:**
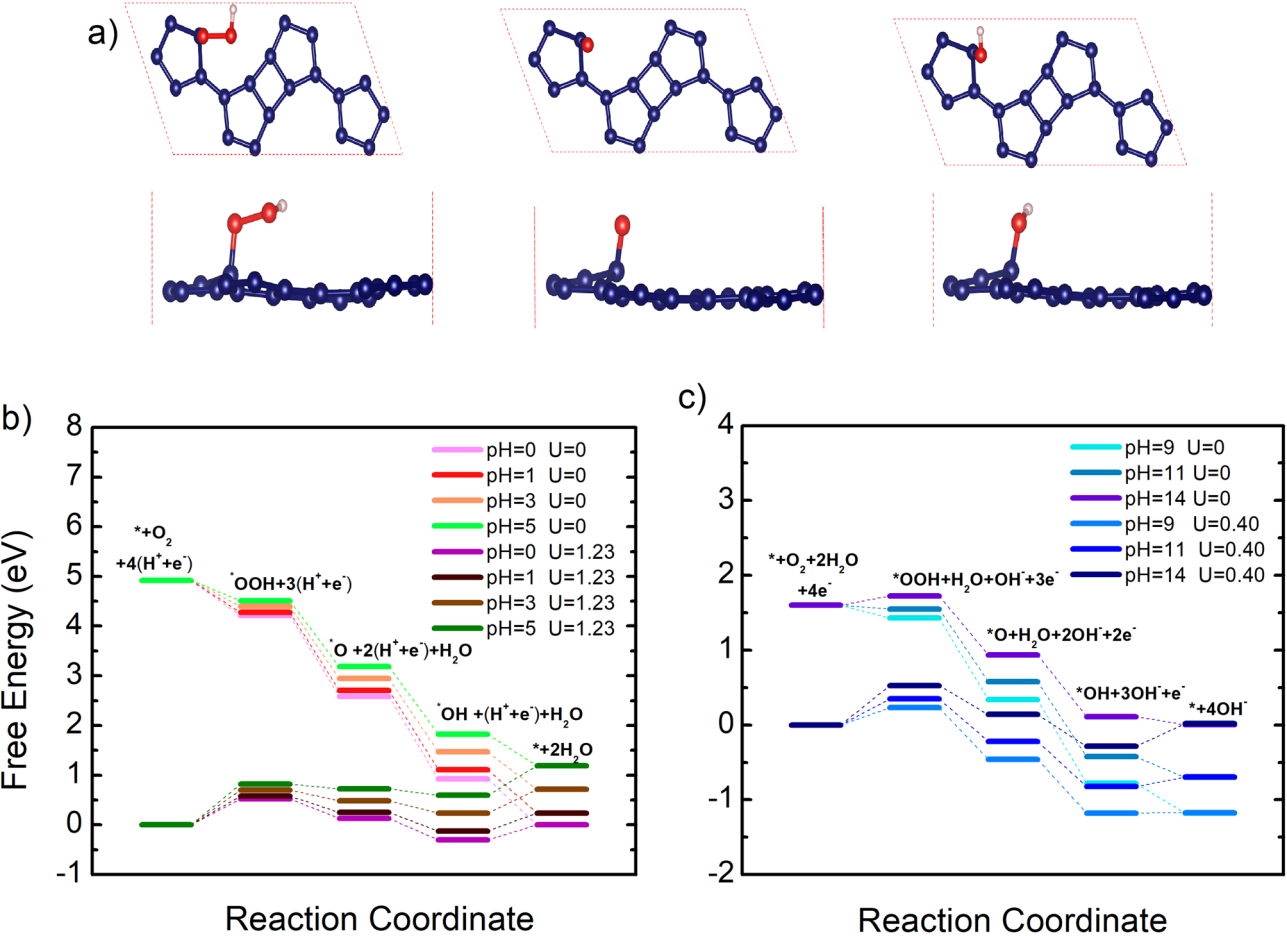
(**a**) Atomic structures of ORR intermediate species (*OOH, *O, and *OH) adsorbed on the GrS. Schematic energy profile for the ORR pathway on the graphsene (**b**) in acidic, and (**c**) in alkaline environment.

In exploring the catalytic potential of GrS for the ORR, the thermodynamics of the ORR processes was carefully analyzed by calculating the Gibbs free energy change (ΔG) for each step along the reaction pathway, providing a thermodynamic perspective on the reaction's viability and efficiency on a GrS catalyst surface. In acidic solutions, the electrode reactions critical to the ORR involve a series of proton and electron transfers leading to water formation: O_2_ + 4(H^+^ + e^−^) → *OOH + 3(H^+^ + e^−^) → *O + 2(H^+^ + e^−^) + H_2_O → *OH + (H^+^ + e^−^) + H_2_O → 2H_2_O, while in alkaline solution that can be expressed as O_2_ + 2H_2_O + 4e^−^ → O + *OOH + H_2_O + OH^−^ + 3e^−^ → *O + 2OH^−^ + H_2_O + 2e^−^ → *OH + 3OH^−^ + e^−^ → 4OH^−^. These reactions illustrate the distinct pathways that the ORR follows in acidic and alkaline solutions, highlighting the importance of understanding these variations in catalytic processes. In acidic conditions, the overall ORR process can be summarized as O_2_ + 4H^+^ + 4e^−^ → 2H_2_O (see Fig. 6). The process initiates with the hydrogenation of the O_2_ molecule, involving the adsorption of a proton and the transfer of an electron, ultimately forming an *OOH species adsorbed on the GrS surface.

The investigation into the oxygen reduction reaction on Graphsene reveals that the initial formation of the *OOH species is energetically favorable, demonstrated by a notable negative free energy change (ΔG) of − 0.706 eV at pH = 0. This finding points to the high reactivity of O_2_ with GrS, initiating the ORR process efficiently. Furthermore, the structural analysis reveals a significant displacement in the carbon atom beneath the adsorbed oxygen species, leading to a buckling effect of approximately 0.48 Å. This structural deformation, illustrated in Fig. 6 for the C_20_OOH configuration, signifies a transition in the electronic structure of GrS from a planar, *sp*^2^-hybridized configuration to a distorted *sp*^*3*^-hybridization, thereby increasing adsorption energies.

Subsequent steps in the ORR, including the formation of the first H_2_O molecule and the further hydrogenation to yield an *OH group, are also exothermic, with the most negative ΔG value (− 0.94 eV) observed at pH = 0. This series of energetically favorable reactions underscores the ORR process's feasibility on GrS at zero potential. However, at the equilibrium potential of 1.23 V, ideally generating 1.23 V per electron to balance the free energy change, both the elementary and final ORR steps on GrS become endothermic, indicating the presence of an overpotential. The rate-determining step (RDS) identified as the formation of *OOH, dictates the ORR's efficiency and U_L_ value. As depicted in Fig. 6, GrS exhibits increasing U_L_ values with pH: 0.52 (pH = 0) < 0.58 (pH = 1) < 0.70 (pH = 3) < 0.82 (pH = 5), demonstrating its optimal catalytic activity at higher pH levels.

In alkaline conditions, the ORR on GrS simplifies to O_2_ + 2H_2_O + 4e^−^ → 4OH^−^. The reaction progresses through an associative proton and electron transfer pathway, starting with *OOH and OH^−^ formation from O_2_ and H_2_O, respectively. This initiates a sequence of reactions leading to *O and subsequently *OH, which ultimately produces OH^−^. Similar to acidic environments, the pH level significantly influences the energy of these transfer steps, affecting the ORR’s efficiency on GrS.

A critical observation from our study is the identification of the O_2_ and H_2_O reaction as the RDS in alkaline conditions, with the energy barrier for this step increasing with the pH level: − 0.52 (pH = 14) < 0.23 (pH = 9) < 0.34 (pH = 11). As illustrated in Fig. 6, a moderately alkaline environment optimizes GrS’s catalytic performance. At an electrode potential of 0.40 V, the detection of endothermic reactions across all steps indicates an overpotential, emphasizing the need for an ideal potential to achieve energetically favorable reactions.

Overall, our findings demonstrate GrS's significant catalytic activity for the ORR across a spectrum of pH conditions, highlighting its potential as a versatile catalyst for electrochemical applications. The detailed analysis of the ORR pathway, especially the impact of pH on the energy levels of proton and electron transfer steps and the implications of the RDS under alkaline conditions, provides valuable insights into optimizing GrS for efficient energy conversion technologies.

## Conclusion

In this study, Graphsene (GrS), a novel graphene allotrope characterized by its unique assembly of tetra-, penta-, and dodeca-carbon rings was introduced and explored. Through rigorous density functional theory calculations, GrS was shown to exhibit remarkable dynamical, mechanical, and thermal stabilities, bringing feasibility to the synthesis of the proposed nanostructure. Additionally, based on cohesive energy study, GrS outperforms several existing carbon-based monolayers in terms of energy. Our investigation into its electronic properties revealed a notable distinction between PBE and HSE06 functionals. While PBE predicted metallic behavior, HSE06 uncovered a small band gap of 20 meV. Due to intrinsic anisotropy in atomic arrangement, distinct mechanical and electronic behavior were observed in zigzag and armchair directions. Furthermore, linear dispersion of frontier bands at M high-symmetry point with Dirac-like cone formation could be expected. Based on ORR processes thermodynamics data, GrS demonstrated exceptional catalytic activity, particularly in acidic conditions, with an essential rate-determining step involving the formation of *OOH. Alkaline conditions revealed the ORR’s dependency on the interaction between O_2_ and H_2_O as the rate-determining step, marking GrS’s capability to maintain substantial catalytic activity despite the inherent challenges of overpotential.

### Supplementary Information

Supplementary Information.

## Data Availability

The datasets used and/or analyzed during the current study are available from the corresponding author on reasonable request.
