# Supplementary material for: Graphsene as a novel porous two-dimensional carbon material for enhanced oxygen reduction electrocatalysis
Source: Sci Rep. 2024 Apr 21;14:9129. doi: 10.1038/s41598-024-59756-3 (PMC11033272; doi:10.1038/s41598-024-59756-3)
Supplement: Supplementary file 1 — Supplementary Information. [file 41598_2024_59756_MOESM1_ESM.docx]

**Electronic Supplementary Information for**

**Graphsene as a Novel Porous Two-Dimensional Carbon Material for Enhanced Oxygen Reduction Electrocatalysis**

**Mohammadreza Hosseini^a^, Maryam Soleimani^b^, Fazel Shojaei^c^, Mahdi Pourfath^b,d*^**

**^a^** Department of Physical Chemistry, Tarbiat Modares University, Tehran, Iran

^b^ School of Electrical and Computer Engineering, University of Tehran, 14395-515 Tehran, Iran

^c^ Department of Chemistry, Faculty of Nano and Bioscience and Technology, Persian Gulf University, Bushehr 75169, Iran

^d^ Institute for Microelectronics, TU Wien, Gußhausstraße 27-29, A-1040, Vienna, Austria

E(θ) = $\frac{C_{11}C_{22}- C_{12}^{2}}{C_{11}\sin^{4}\left( \theta\right)+\left[ \frac{\left( C_{11}C_{22}- C_{12}^{2} \right)}{C_{66}}-2C_{12} \right]\sin^{2}\left( \theta\right)\cos^{2}\left( \theta\right)+C_{22}\cos^{4}\left( \theta\right)}$ (S1)

ν(θ) = $\frac{C_{12}\sin^{4}\left( \theta\right)- \left[ C_{11}+C_{22}-\frac{\left( C_{11}C_{22}- C_{12}^{2} \right)}{C_{66}} \right]\sin^{2}\left( \theta\right)\cos^{2}\left( \theta\right)+C_{12}\cos^{4}\left( \theta\right)}{C_{11}\sin^{4}\left( \theta\right)+\left[ \frac{\left( C_{11}C_{22}- C_{12}^{2} \right)}{C_{66}}-2C_{12} \right]\sin^{2}\left( \theta\right)\cos^{2}\left( \theta\right)+C_{22}\cos^{4}\left( \theta\right)}$ (S2)

| 79.510 | 21.084 | 0.000 | 0.000 | 0.000 | 0.000 |
| --- | --- | --- | --- | --- | --- |
| 21.084 | 187.362 | 0.035 | 0.000 | 0.000 | 0.000 |
| 0.000 | -0.035 | 0.182 | 0.000 | 0.000 | 0.000 |
| 0.000 | 0.000 | 0.000 | 0.144 | 0.000 | 0.000 |
| 0.000 | 0.000 | 0.000 | 0.000 | 0.112 | 0.000 |
| 0.000 | 0.000 | 0.000 | 0.000 | 0.000 | 27.578 |

Table S1. GrS 6×6 stiffness tensor in the unit of GPa
